# The Relationship Between Acute-Phase Circuit Occlusion and Blood Calcium Concentration in an Ex Vivo Continuous Renal Replacement Therapy Model

**DOI:** 10.7759/cureus.59330

**Published:** 2024-04-30

**Authors:** Yu Kashima, Hiroyuki Koami, Yuichiro Sakamoto

**Affiliations:** 1 Medical Engineering, Junshin Gakuen University, Fukuoka, JPN; 2 Department of Emergency and Critical Care Medicine, Saga University, Saga, JPN

**Keywords:** ex vivo model, thrombotic occlusion, chelate, citrate, continuous renal replacement therapy

## Abstract

Background and objective

Continuous renal replacement therapy (CRRT) is a blood purification therapy modality for the treatment of renal failure in critically ill hospitalized patients with multiorgan dysfunction, effectively preventing uremia and multiple organ failure while improving renal function. However, the perfusion of patient blood through extracorporeal circulation often results in unexpected early occlusion of the CRRT circuit or hemofilter, leading to frequent interruptions in CRRT and wastage of medical resources. Moreover, clinical research on such circuit occlusions is limited. In Japan, CRRT circuits require long-term perfusion, often lasting 24 hours or more, indicating the need for a model capable of inducing occlusion at any arbitrary time; this model can evaluate various aspects, including causes and underlying mechanisms, and contribute to the development of an occlusion prediction method. Hence, we hypothesized the need for a model for inducing occlusion at arbitrary time points. Consequently, we strove to develop an *ex vivo* circuit occlusion model involving the injection of calcium into circulating citrated animal blood to evaluate the relationship between the amount of calcium chloride injected, circuit occlusion time, and changes in circuit pressure over time.

Methods

We developed a circuit occlusion model using a commercially available CRRT circuit, polysulfone membrane hemofilter, heating extension tube, and thermostatic water bath, along with commercially available citrated bovine whole blood. The circuit was filled with blood over a 10-min duration using a roller pump and was occluded after a specific period by varying the flow rate of calcium injected into bovine whole blood. Additionally, continuous injection of 1 mEq/mL calcium chloride into the circuit was maintained while bovine whole blood circulated. Measurements were performed at each calcium injection flow rate (2, 3, and 4 mL/h), with each measurement performed five times. The group that did not receive calcium injection was used as the control (0 mL/h: Con), and the experiment was performed three times. Groups were defined as "0, 2, 3, and 4" for each calcium injection flow rate. The relationship among the amount of calcium chloride injected, circuit occlusion time, and changes in circuit pressure over time was evaluated. Furthermore, blood tests and blood viscoelastic tests were performed at arbitrary times.

Results

The circuit occlusion time varied with each calcium injection flow rate, and a significant difference was observed between each group (p<0.05). Circuit pressure gradually changed at four min before occlusion when calcium was injected at 2, 3, and 4 mL/h, with a more rapid change at one min before occlusion. We measured circuit pressure at four and one min before occlusion (-4 min, and -1 min, respectively), and at the time of circuit occlusion (0 min) in the Con and 4 mL/h groups. Significant differences were observed in AP between -4 min and 0 min and -1 min and 0 min at a calcium flow rate of 4 mL/h. Additionally, significant differences were seen in prefilter and return pressures between -4 min and 0 min, -4 min and -1 min, and -1 min and 0 min at a calcium flow rate of 4 mL/h (p<0.05).

Conclusions

Our proposed model accurately estimated the occlusion time based on changes in circuit pressure. This model can be used to create various experimental systems depending on the desired occlusion time.

## Introduction

Acute kidney injury can complicate various critical illnesses, including sepsis, and patients have a poor prognosis even after undergoing multidisciplinary treatment in the ICU [1]. Blood purification therapy is employed when urine output decreases and renal function worsens. Continuous renal replacement therapy (CRRT) gradually removes uremic substances, even in pathological conditions with unstable hemodynamics. It corrects electrolyte and acid-base balance and controls water balance. Furthermore, it prevents uremia and multiple organ failure and improves renal function [2,3]. Recently, specialized hemofilters that adsorb inflammatory substances (such as cytokines, which play a central role in exacerbating sepsis) have been clinically used [4] and these devices are expected to improve clinical outcomes.

However, the CRRT circuit or hemofilter often experiences unexpected early occlusion owing to perfusion of the patient’s blood through extracorporeal circulation [5]. Especially, occlusion due to thrombus formation necessitates the replacement of the entire CRRT circuit. Such unexpected discontinuation of CRRT results in the wastage of medical resources, including disposal of blood from patients under intensive care in unstable conditions, replacement of medical consumables such as circuits, and allocation of medical staff (including clinical engineers) who must urgently replace circuits [6]. Moreover, clinical research on such unexpected early circuit occlusions is limited [5,7,8].

In Japan, CRRT circuits require long-term perfusion, often extending for 24 hours or more [9,10]. Therefore, we hypothesized the necessity of a model capable of inducing occlusion at any time point. Citric acid was added to commercially available animal blood, and calcium was injected into the circulating animal blood. This model can evaluate various aspects, such as the causes and underlying mechanisms, and aid in the development of an occlusion prediction method. In this study, we aimed to develop an *ex vivo* circuit occlusion model to evaluate the relationship between the amount of calcium chloride injected, circuit occlusion time, and changes in circuit pressure over time.

## Materials and methods

Blood preparation

We obtained 1000 mL of commercially available citrated bovine whole blood (DARD Co. Ltd., Tokyo, Japan), which was used within two days (final citric acid concentration: 0.5%).

Ex vivo CRRT model experimental circuit configuration

**Figure 1:**
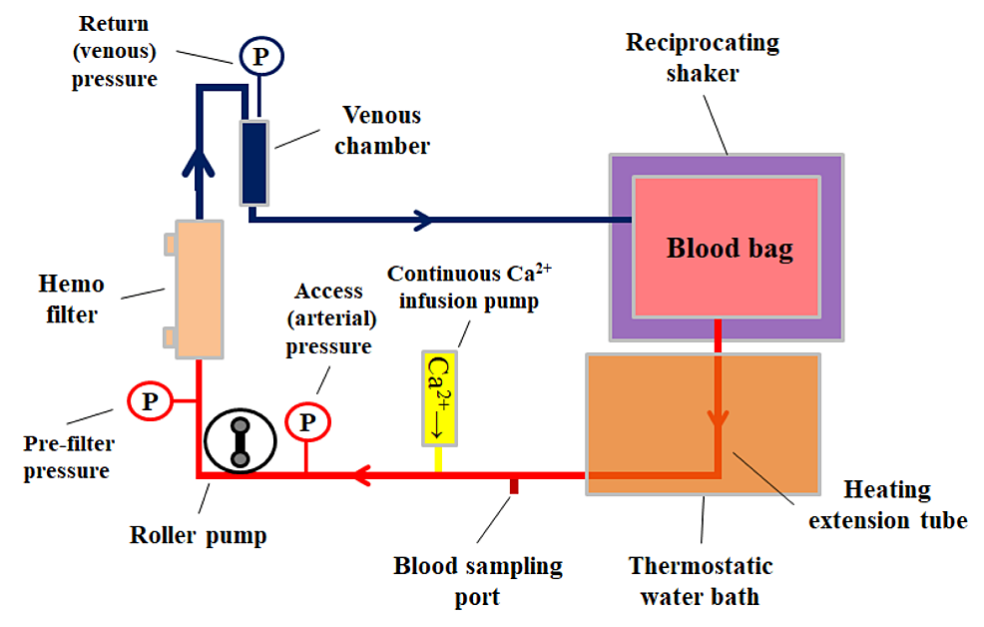
Experimental circuit Calcium chloride correction solution (1 mEq/mL) was continuously injected into the circuit during blood circulation. Circuit pressure was measured using a continuous renal replacement therapy device, and blood was collected for testing from the sampling port on the access side of the circuit. A heating tube was connected to the access side of the circuit, and the blood was placed in a thermostatic water bath set at 39 °C Image credit: Yu Kashima

A commercially available CRRT circuit (CHDF-FS, Asahi Kasei Medical Co., Ltd., Tokyo, Japan) was connected to a polysulfone membrane hemofilter (AEF-13, Asahi Kasei Medical Co., Ltd., Japan) and primed with saline. A heating extension tube was connected to the access side of the circuit and primed with saline, and a heating extension tube was immersed in a thermostatic water bath (SP-12N, TAITEC Corporation, Kyoto, Japan) set at 39 °C. A blood bag (SENKO Medical Instrument Mfg. Co., Ltd., Tokyo, Japan) was filled with 500 mL of citrated bovine whole blood, previously refrigerated at 14 °C, and any air within the blood bag was removed. Subsequently, the bag was connected to the primed circuit, and the blood pump was activated, simultaneously displacing 150 mL of saline with blood in the CRRT circuit. The circuit was filled with blood over a 10-min duration using a roller pump. The blood bag was regularly shaken using a Maxi Rocker Model 4638 shaker (Barnstead Lab-Line, Ramsey, MN). Concurrently, a continuous injection of 1 mEq/mL of calcium chloride correction solution (Otsuka Pharmaceutical Factory, Inc., Tokyo, Japan) into the circuit was maintained while blood circulated. Circuit pressure was measured using a CRRT device (ACH-Σ, Asahi Kasei Medical Co., Ltd.), and blood samples were collected from the access side of the circuit via a sampling port (Figure 1).

Categories for CRRT measurement

Calcium administration commenced at 0 min, and blood samples were obtained at arbitrary times (0, 60, 120, 180, 240, 300, and 360 min). Furthermore, blood tests and blood viscoelastic tests were performed. The blood tests included white blood count (WBC), platelet (PLT), hemoglobin (HGB), C-reactive protein (CRP), serum total calcium (T-Ca), and albumin (ALB). Additionally, each factor [including Citrated Kaolin (CK)_R and CK_ Maximum amplitude (MA)] of the whole blood viscoelastic test TEG®6s (TEG) (Haemonetics Corp., Boston, MA) was assessed. CK is a standard TEG reagent that requires kaolin for the activation of coagulation. The coagulation profile resulting from kaolin activation indicates the time to first measurable clot formation, the kinetics of clot formation, clot strength, and clot breakdown (fibrinolysis). “R” (unit: min) is the time elapsed from the start of the test until sufficient resistance is created by clotting, resulting in a measured pin amplitude of 2 mm. This parameter corresponds to the initiation phase of clotting caused by enzymatic clotting factors. A long R indicates slow clot formation. MA (unit: mm) refers to the point at which clot strength reaches its maximum value, reflecting the formation of platelet-fibrin cross-links via GPIIb/IIIa receptors [11].

Circuit pressure was continuously assessed using the following parameters: access pressure (AP), denoting the pressure preceding the pump; prefilter pressure (PFP), signifying the pressure anterior to the hemofilter; return pressure (RP), representing the pressure within the venous chamber (V-chamber); and transmembrane pressure (TMP). Measurements were performed at each calcium injection flow rate (2, 3, and 4 mL/h), with each measurement performed five times. The case where calcium was not injected was used as the control (0 mL/h: Con), and the experiment was performed three times. Groups were defined as "0, 2, 3, and 4" for each calcium injection flow rate. The circuit was considered occluded when PFP or RP exceeded 100 mmHg, and the experiment was terminated. The maximum perfusion time was set at 360 min, and the experiment was terminated if circuit occlusion did not occur.

The CRRT flow settings were configured in continuous hemodialysis (CHD) mode with a blood flow rate of 100 mL/min and a dialysate flow rate of 10 mL/h. Saline was selected as the dialysate. These parameters were set as the lowest feasible flow rates of CHD since ACH-Σ requires the selection of the blood purification mode owing to its safety mechanism.

Statistical analysis

The relationship between each calcium injection rate and circuit occlusion time, changes in circuit pressure leading to circuit occlusion, and changes in calcium injection flow rate and blood test values were analyzed. Circuit pressure, the pressure at the beginning of CRRT (designated as 0 mmHg), and any changes in circuit pressure at any given time were recorded.

Data are presented as median and interquartile range (IQR) for continuous variables. Outcome measures of interest (occlusion time, circuit pressure, and blood-tested values) were compared among study groups. Data were compared using the Kruskal-Wallis test for continuous variables. In all analyses, statistical significance was set at a two-tailed test with p<0.05. Statistical analyses were performed using JMP® Pro version 17.0.0 (SAS Institute Inc., Cary, NC).

## Results

Calcium injection flow rate and occlusion time

**Figure 2:**
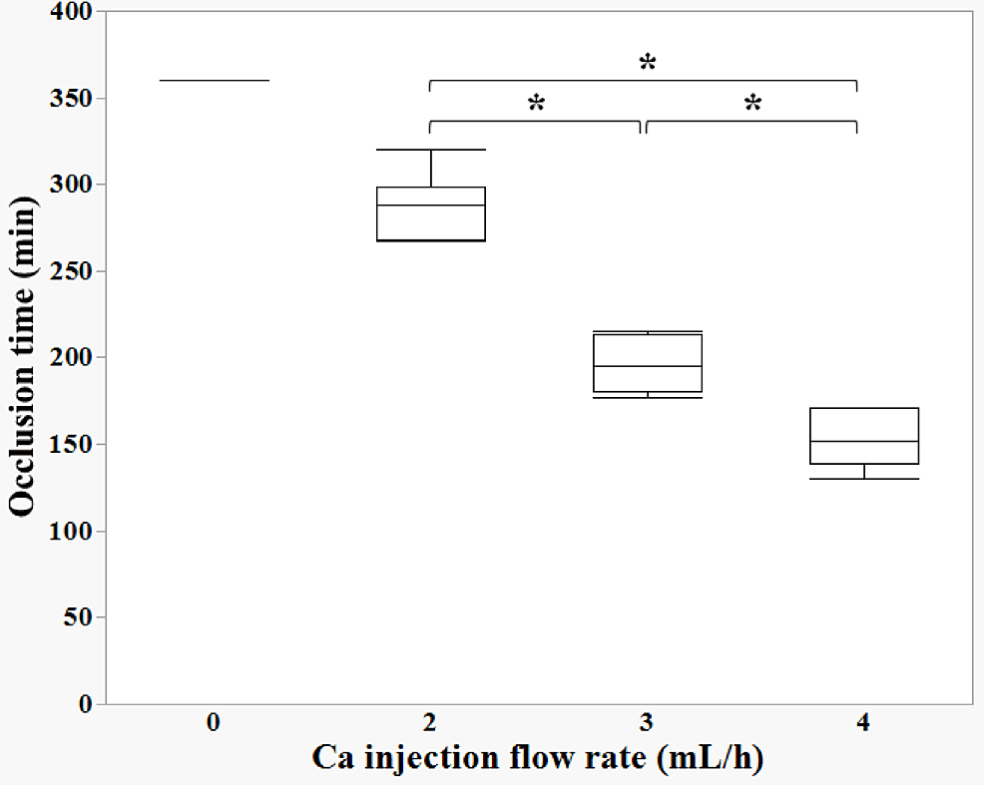
Relationship between calcium infusion rate and occlusion time A decrease in the circuit occlusion time is observed with increasing calcium injection flow rate. In contrast, in the control (0 mL/h: Con) group, occlusion did not occur after 360 min (*p<0.05) Data were compared using the Kruskal–Wallis test for continuous variables (significance level: p=0.05)

Circuit occlusion time varied with each calcium injection flow rate, and a significant difference was observed between each group (Figure 2). Additionally, decreased circuit occlusion times were noted with higher calcium injection flow rates. In contrast, the Con group did not undergo occlusion after 360 min.

Changes in circuit pressure until circuit occlusion

**Figure 3:**
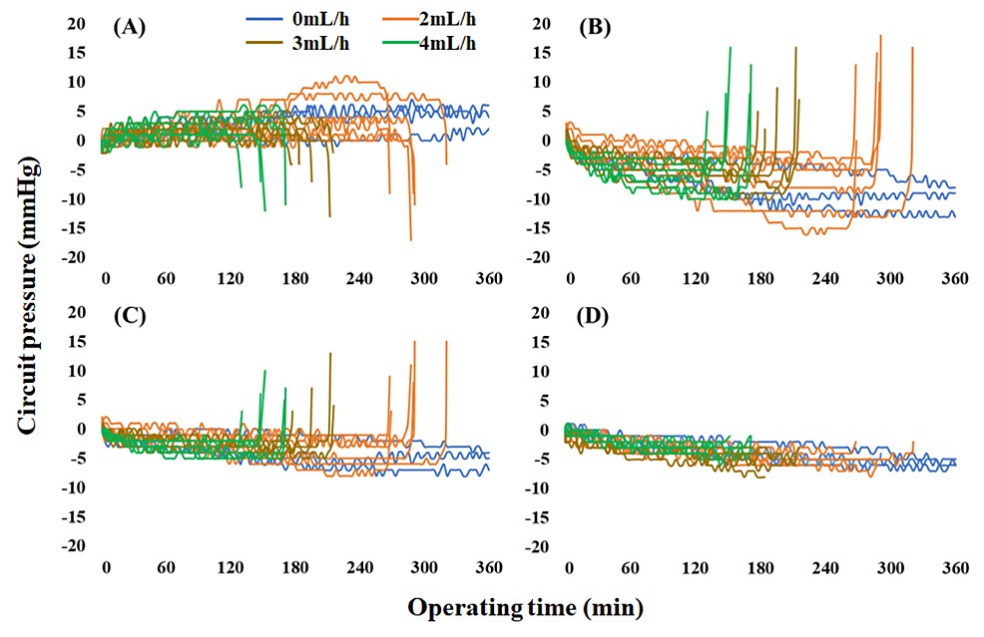
Changes in circuit pressure over time as a function of the calcium injection flow rate (A) shows access pressure, (B) shows prefilter pressure, (C) shows return pressure, and (D) shows transmembrane pressure. Access pressure gradually increased over time and rapidly decreased before occlusion when calcium was injected at 2, 3, and 4 mL/h. However, prefilter pressure and return pressure gradually decreased over time and sharply increased just before occlusion. Transmembrane pressure decreased in all cases; however, no changes were observed owing to occlusion

AP gradually increased over time and rapidly decreased before occlusion when calcium was injected at 2, 3, and 4 mL/h (Figure 3). However, PFP and RP gradually decreased over time and sharply increased just before occlusion. TMP decreased in all cases; however, no changes were observed owing to occlusion. An analysis of the changes in circuit pressure in 10-s increments from 10 min before circuit occlusion to occlusion revealed that circuit pressure gradually changed at four min before occlusion when calcium was injected at 2, 3, and 4 mL/h, with a more rapid change at one min before occlusion.

**Figure 4:**
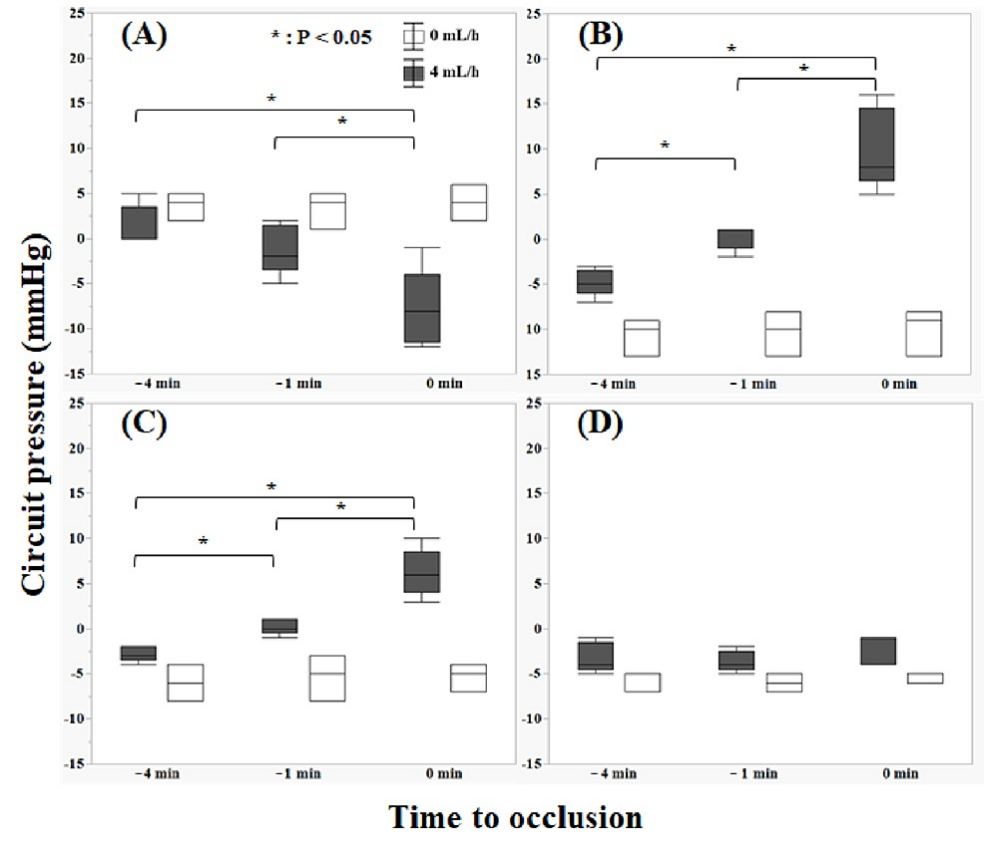
Pressure changes in each circuit just before occlusion (0 mL/h: Con, 4 mL/h) (A) shows access pressure, (B) shows prefilter pressure, (C) shows return pressure, and (D) shows transmembrane pressure. Circuit pressure was measured four min before occlusion (–4 min), one min before occlusion (–1 min), and at the time of circuit occlusion (0 min). Significant differences were observed in AP between –4 min and 0 min and –1 min and 0 min at a calcium flow rate of 4 mL/h. Additionally, significant differences were observed in PFP and RP between –4 min and 0 min, –4 min and –1 min, and –1 min and 0 min at a calcium flow rate of 4 mL/h Data were compared using the Kruskal–Wallis test for continuous variables (significance level: p=0.05)

Therefore, we measured circuit pressure four min before occlusion (-4 min), one min before occlusion (-1 min), and at the time of circuit occlusion (0 min) in the Con and 4 mL/h groups. Significant differences were observed in AP between -4 min and 0 min and -1 min and 0 min at a calcium flow rate of 4 mL/h (Figure 4). Additionally, significant differences were observed in PFP and RP between -4 min and 0 min, -4 min and -1 min, and -1 min and 0 min at a calcium flow rate of 4 mL/h.

Changes in calcium injection flow rate and blood-tested values

**Figure 5:**
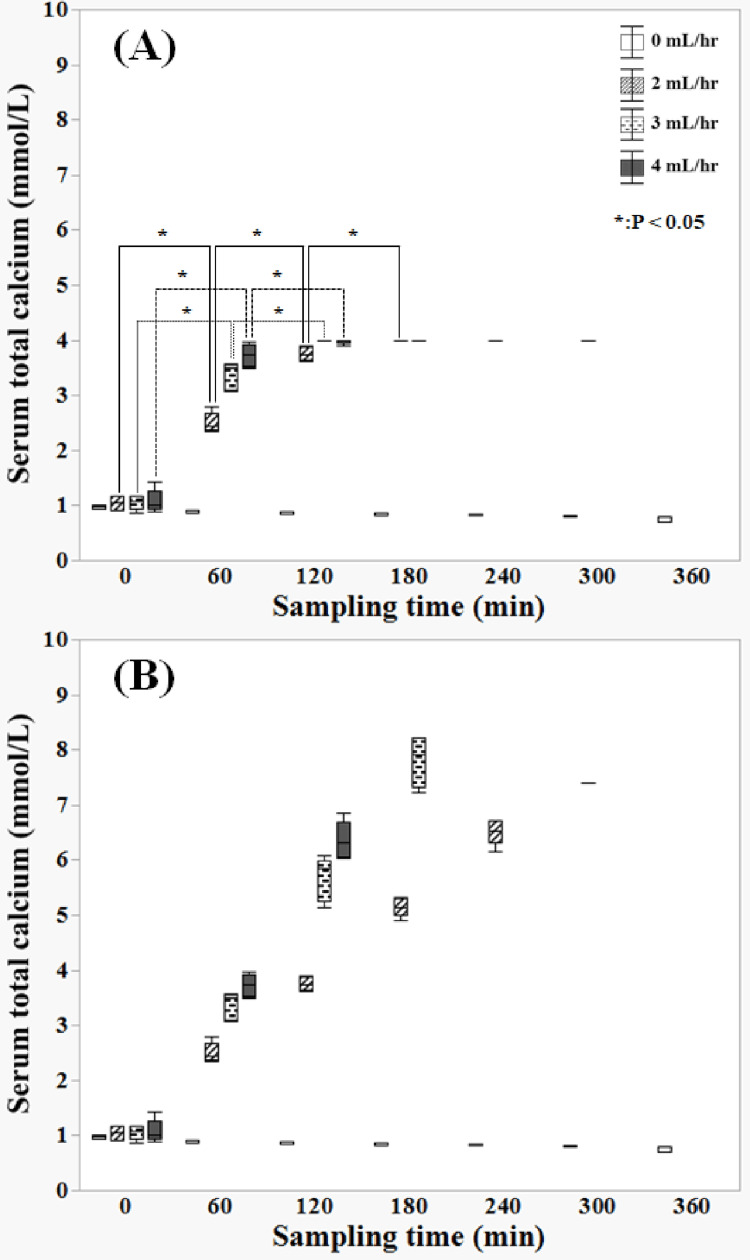
Changes over time in total serum calcium (A) shows the changes over time in the actual value of total serum calcium (measurement upper limit: 4 mmol/L). Significant differences were observed between the 0–60, 60–120, and 120–180 min groups at a calcium flow rate of 2 mL/h. Significant differences were observed between the 0–60 and 60–120 min groups at calcium flow rates of 3 and 4 mL/h. Measurements exceeding 4 mmol/L were not possible owing to the upper limit of calcium measurement. (B) shows the changes over time in (A), plus over 4 mmol/L including estimated values. Values exceeding 4 mmol/L, or more were calculated as estimated values, as the increase in calcium was linear Data were compared using the Kruskal–Wallis test for continuous variables (significance level: p=0.05)

Significant differences were observed in the T-Ca levels between the 0-60, 60-120, and 120-180 min groups at a calcium flow rate of 2 mL/h (Figure 5A). Significant differences were observed between the 0-60 and 60-120 min groups at calcium flow rates of 3 and 4 mL/h. Measurements exceeding 4 mmol/L were not possible owing to the upper limit of calcium measurement. Therefore, values exceeding 4 mmol/L were calculated as estimated values since the increase in calcium was linear (Figure 5B).

**Figure 6:**
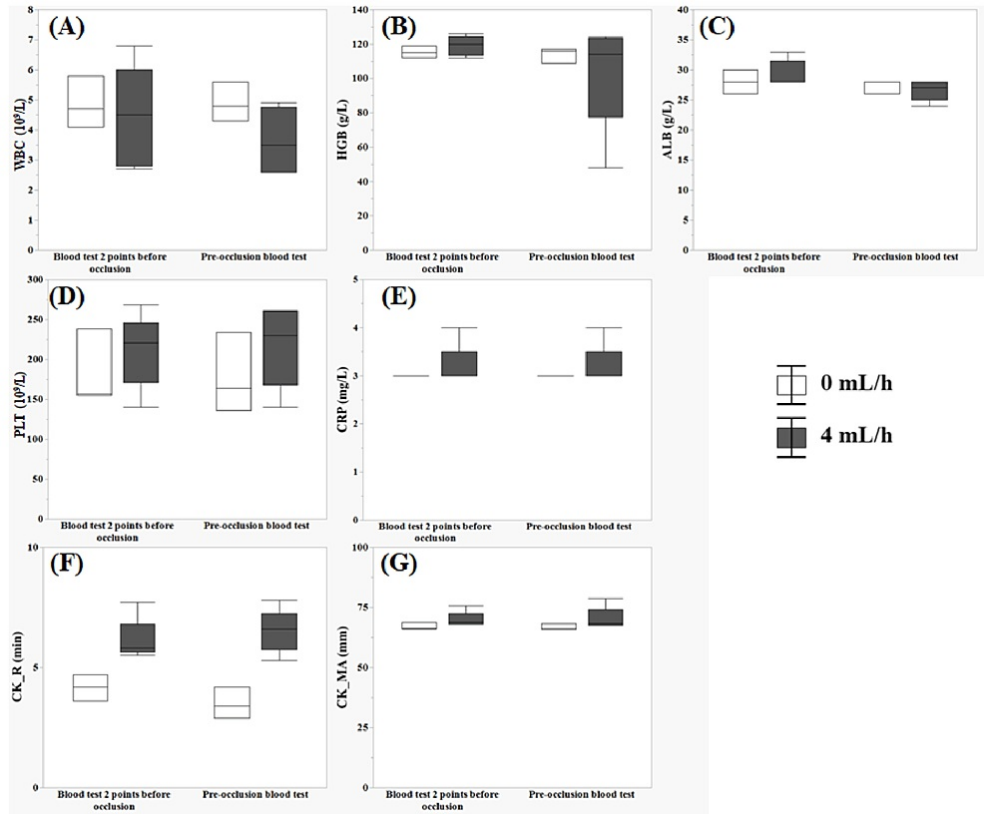
Blood test for the two most recent points of circuit occlusion (A) shows WBC, (B) shows HGB, (C) shows ALB, (D) shows PLT, (E) shows CRP, (F) shows CK_R, and (G) shows CK_MA No significant differences were observed between the blood tests performed two points before occlusion and those performed pre-occlusion (p>0.05). CK is a standard TEG reagent that requires kaolin for activation of coagulation. The coagulation profile resulting from kaolin activation indicates the time to first measurable clot formation, the kinetics of clot formation, clot strength, and clot breakdown (fibrinolysis). “R” (unit: min) is the time elapsed from the start of the test until sufficient resistance is created by clotting, resulting in a measured pin amplitude of 2 mm. This parameter corresponds to the initiation phase of clotting caused by enzymatic clotting factors. A long R indicates slow clot formation. MA (unit: mm) refers to the point at which clot strength reaches its maximum value, reflecting the formation of platelet-fibrin cross-links via GPIIb/IIIa receptors Data were compared using the Kruskal–Wallis test for continuous variables (significance level: p=0.05) WBC: white blood cell; HGB: hemoglobin; ALB: albumin; PLT: platelet; CRP: C-reactive protein; CK_R: Citrated Kaolin _R; CK_MA: Citrated Kaolin _Maximum amplitude

Figure 6 illustrates the changes in WBC, HGB, ALB, PLT, CRP, CK_R, and CK_MA at the two most recent points before circuit occlusion in the Con group and at calcium flow rates of 4 mL/h. No significant differences were observed between the blood tests performed two points before occlusion and those performed before occlusion (p>0.05).

## Discussion

In this study, we developed a reproducible model with occlusion of the CRRT circuit after a specific period by varying the flow rate of calcium injection into bovine whole blood. Our model demonstrated that the circuit pressure gradually changed at four min before occlusion when calcium was injected at 2, 3, and 4 mL/h, with a more rapid change at one min before occlusion. The results highlight the possibility of inducing circuit occlusion for each calcium injection flow rate at a specific duration. We believe this study will have a significant impact as it is the first of its kind to develop an *ex vivo* circuit occlusion model.

**Figure 7:**
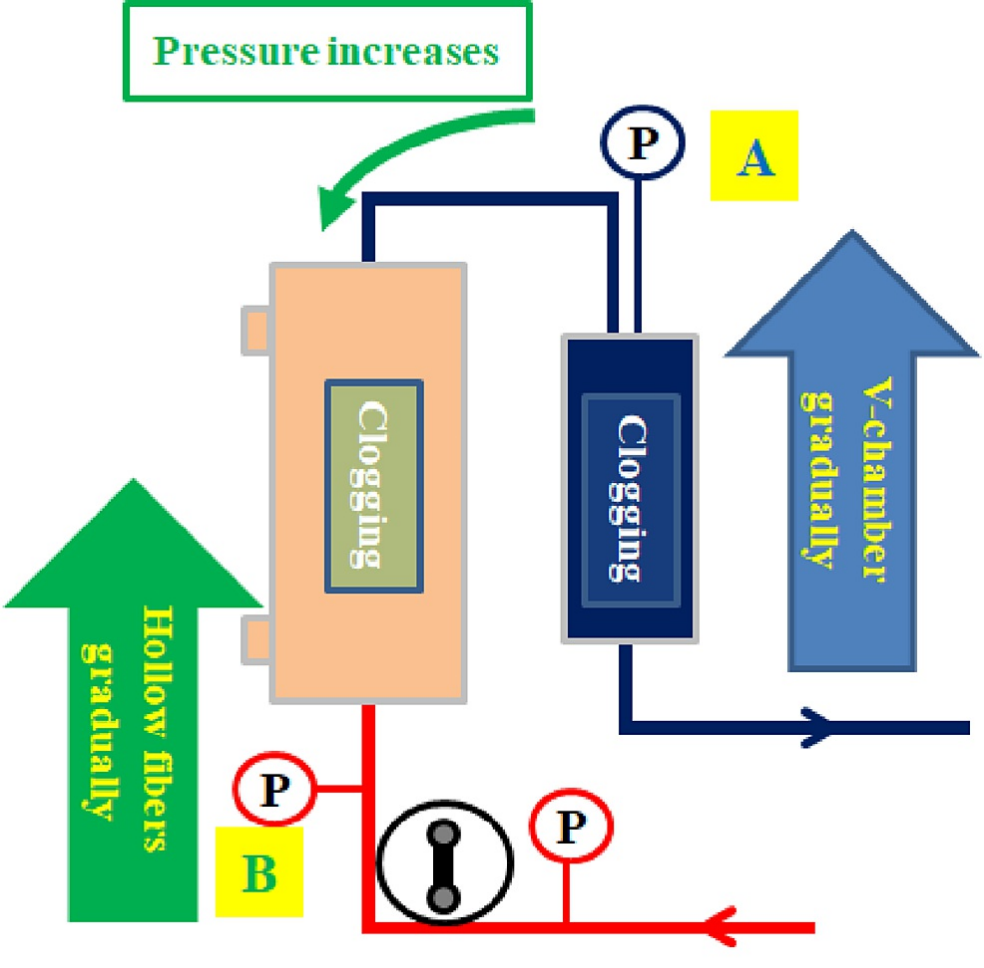
Mechanism of increase in circuit pressure Gradual clogging of the V-chamber → gradual increase in return pressure (RP)→ V-chamber complete occlusion→ RP rapidly increases (A). As the V-chamber becomes clogged, the hollow fibers of the hemofilter gradually become clogged→ the RP and prefilter pressure (PFP) rapidly increase (B), causing perfusion failure and complete occlusion Image credit: Yu Kashima

In our *ex vivo* model, the continuous infusion of calcium caused thrombus formation in the venous chamber, progressively increasing RP and PFP. Triplicate assays confirmed that the circuit was occluded. AP gradually increased, whereas PFP and RP gradually decreased as CRRT commenced. Blood viscosity increased to 50-300% when blood temperature decreased from 37 ℃ to 22℃ [12-14]. Moreover, bovine blood (previously refrigerated at 14 ℃) was heated by perfusion, and the viscosity of the blood gradually decreased. At a calcium flow rate of 4 mL/h, PFP and RP significantly increased between -4 min and -1 min and between -1 min and 0 min. PFP and RP sharply increased at -1 min before occlusion, suggesting a strong responsiveness to occlusion. We focused on the pressure change (∆P) between -4 min and -1 min and between -1 min and 0 min using the median values of PFP and RP, which helped describe the coagulation changes that occurred in the circuit. The V-chamber gradually became clogged, leading to a gradual increase in RP. Over time, the V-chamber became completely occluded, causing RP to rapidly increase. Simultaneously, PFP increased owing to clogging within the hollow fibers of the hemofilter. RP and PFP rapidly increased within 1 min, causing perfusion failure and complete occlusion. Consequently, returning the remaining blood in the circuit became impossible (Figure 7). In other words, calcium chelated sodium citrate, and the clotting reaction was possibly accelerated in the V-chamber and hemofilter, where blood tends to stagnate [15-17].

**Figure 8:**
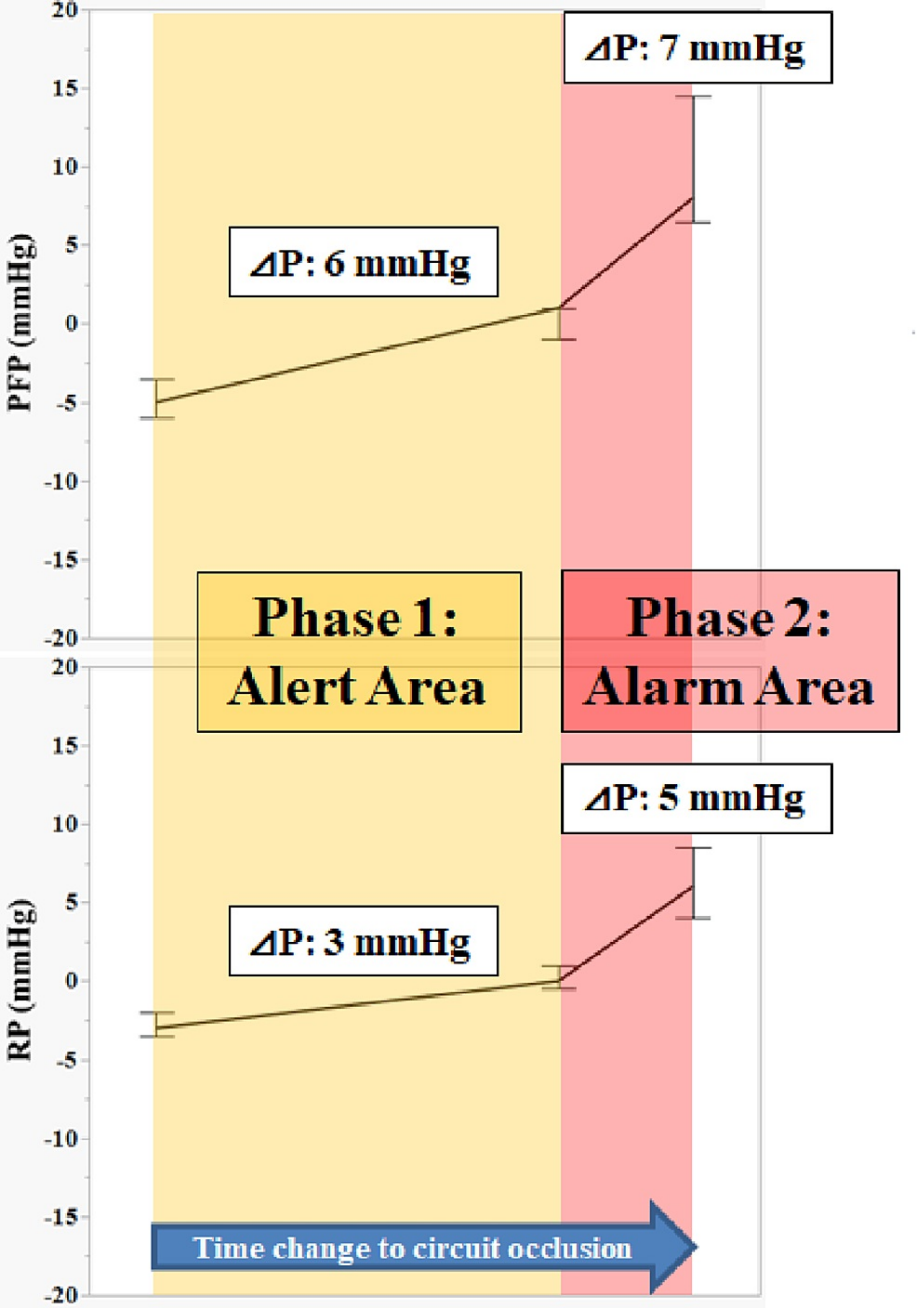
Clinical circuit pressure alarm strategy Phase 1: Alert, prefilter pressure (PFP) ∆6 mmHg and return pressure (RP) ∆3 mmHg→ V-chamber clogging; PFP ∆6 mmHg only→ Hemofilter clogging. Prepare to return the blood remaining in the circuit. Phase 2: Alarm, PFP ∆6+∆7 mmHg and RP ∆3+∆5 mmHg→ V-chamber occlusion; PFP ∆6+∆7 mmHg only→ Hemofilter occlusion. It is imperative to commence preparation to immediately return the remaining blood in the circuit Image credit: Yu Kashima

The slight change between Z-4 min and -1 min in the short-term occlusion model of this study indicates a sign of a sudden pressure change just before occlusion. The change from -1 min to 0 min can be considered the final change before complete occlusion. This result is assumed to represent changes in circuit pressure in clinical practice. Therefore, changes in circuit pressure may be used as various indicators, for example, as a predictor of occlusion time and as a determinator of CRRT termination time. Based on the results of this study, we devised a clinical circuit pressure alarm strategy and proposed a method to estimate this based on changes in circuit pressure (Figure 8). For example, we assumed that PFP and RP increase by 6 and 3 mmHg between 30 and 60 min, respectively (Phase 1). PFP and RP will rapidly increase within 30 min, and the V-chamber and hemofilter will most likely become occluded, given the suspected thrombus formation in the V-chamber (Phase 2). Alternatively, if only PFP increases by 6 mmHg between 30 and 60 min (Phase 1), it raises suspicions of hemofilter occlusion, with a high probability that PFP will rapidly increase within 30 min and the circuit will become occluded (Phase 2). In either case, early preparation to initiate blood return within the circuit is imperative. This slight change can be displayed as an alarm on the CRRT device to assist in monitoring operators.

Additionally, this model can be used to create different experimental systems depending on the occlusion time. For example, the effects of CRRT mode (continuous hemofiltration vs. continuous hemodiafiltration) and differences in hemofilter membrane material [polysulfone vs. polyacrylonitrile-co-sodium methallyl sulfonate copolymer (AN69)] on occlusion time can be studied using a model wherein calcium is injected at 2 mL/h. This finding provides a basis for future research. However, this study could not quantify the exact amount of change owing to the small number of parameters. Increasing the number of parameters could enhance the ability to detect internal pressure changes (∆P) within the circuit more accurately. Predicting occlusion based on the slope of the amount of change might be possible.

Calcium concentration linearly increased at calcium flow rates of 2, 3, and 4 mL/h. Injecting calcium into the circuit leads to increased calcium concentration, indicating the chelation of sodium citrate and resulting in circuit occlusion. A calcium concentration of 6.5 mmol/L [(6.3-6.7: 240 min) (median (IQR)], 7.9 mmol/L [(7.3-8.2: 180 min) (median (IQR)], and 6.3 mmol/L [(6.1-6.7: 120 min) (median (IQR)] was observed at a calcium flow rate of 2 mL/h, 3 mL/h, and 4 mL/h, respectively (Figure 5B). The estimated calcium concentration (180 min) reached 7.9 mmol/L at a calcium flow rate of 3 mL/h and occlusion time of 195 min. Therefore, a calcium concentration that exceeds 7.5 mmol/L marks the limit at which the circuit is suddenly occluded. It is an estimate and is significant because it may indicate excessive calcium administration during regional citrate anticoagulation [18,19].

No significant changes were observed in the inflammatory response (WBC and CRP), dilution or hemolysis (HGB) caused by calcium injection, or coagulation response (PLT) caused by platelet aggregation in our model. This indicates that injecting calcium had almost no effect on other factors. However, this blood test does not reflect the results immediately before the circuit was occluded. Therefore, the influence of circuit coagulation cannot be completely excluded. No significant difference was observed between the blood test performed two points before occlusion and that performed before occlusion for ALB (Figure 6), although a trend towards a decrease at all calcium injection flow rate groups was observed. This is likely attributed to an adsorption reaction of ALB on polyvinyl chloride, which is used in blood circuits and seat bags [20,21]. Additionally, polyvinyl chloride transports small amounts of CO_2_ gas [22-23], which might have affected the pH of the blood, leading to charge changes and ALB measurement errors. These effects may be corrected in humans and animals via respiration and metabolism. However, this study uses an *ex vivo* blood circulation model, and one of its disadvantages is that it does not affect metabolism.

The changes in each TEG factor were monitored as coagulation parameters; however, no consistent trend was observed. Similar to blood tests, it does not reflect the results immediately before the circuit is occluded. Therefore, the influence of circuit coagulation cannot be completely excluded. However, this monitoring approach was ineffective in this study. Consequently, this monitoring approach does not reflect results immediately before circuit occlusion and is not a research model that simulates sepsis. It may be an effective indicator in experimental systems that simulate pathological conditions such as sepsis that cause coagulation and fibrinolysis abnormalities [24,25].

We proposed a clinical circuit pressure alarm strategy estimated based on changes in circuit pressure. If there is no vascular access failure, predicting thrombus formation within the circuit could potentially be facilitated by programming pressure changes per unit time into a CRRT device. Drawing from the previous example, if PFP increases by 6 mmHg and RP increases by 3 mmHg in 60 min, the CRRT device could display an alarm message such as “Suspected V-chamber thrombus formation” on the screen for CRRT supervisors, along with suggested countermeasures. Although the detailed technology depends on the technical capabilities of the CRRT manufacturer, this technique can be achieved. Furthermore, allowing for arbitrary changes in the ∆P setting of PFP and RP would enhance its versatility and enable it to respond to changing environmental factors (such as blood flow rate and hemofilter type).

A limitation of this study was that it was conducted using commercially available bovine blood, which is biologically different from human blood. Therefore, similar experiments using human blood may not yield similar results. However, ethical and economic issues exist with using human blood, while using blood from other animals in similar studies remains controversial [26]. As this study was an *ex vivo* evaluation, it could not reflect hematologic changes caused by the respiratory state of the living body and metabolic reactions. Conditions such as coagulation, fibrinolysis, and inflammation change momentarily in the living body. Nevertheless, the study's advantage lies in its ability to independently evaluate blood reactions, excluding biological reactions. When evaluating different biological responses, the challenge lies in evaluating and analyzing them through *ex vivo* experiments and clinical data.

Our proposed clinical circuit pressure alarm strategy originates from a short-time circuit occlusion model. Therefore, the timeframe differs from that of clinical CRRT, which assumes long-term driving. Comparing and analyzing ∆P obtained from this study with clinical data may aid the construction of a more accurate alarm strategy based on changes in ∆P per unit time. This is an area we intend to explore in the future.

## Conclusions

We developed a reproducible model wherein the CRRT circuit was occluded after a specific period by varying the calcium injection flow rate into citrated bovine whole blood. Further research is needed to increase and verify the dataset to predict occlusion based on changes in circuit pressure. However, this study potentially contributes to improving the quality of clinical evaluation, securing medical safety, and averting economic losses in healthcare. Furthermore, creating a model that reproduces sepsis and conducting studies aimed at preventing acute-phase circuit occlusion resulting from inflammation-induced coagulopathy are necessary in the future.
